# The Association between Sleep Duration and Quality with Readmissions: An Exploratory Pilot-Study among Cardiology Inpatients

**DOI:** 10.3390/clockssleep2020011

**Published:** 2020-04-02

**Authors:** Clementine Labrosciano, Rosanna Tavella, Amy Reynolds, Tracy Air, John F. Beltrame, Isuru Ranasinghe, Robert J. T. Adams

**Affiliations:** 1 Adelaide Medical School, University of Adelaide, The Queen Elizabeth Hospital Campus, Woodville, SA 5011, Australia; rosanna.tavella@adelaide.edu.au (R.T.); tracy.air@adelaide.edu.au (T.A.); john.beltrame@adelaide.edu.au (J.F.B.); isuru.ranasinghe@adelaide.edu.au (I.R.); 2 Basil Hetzel Institute for Translational Health Research, Adelaide, SA 5011, Australia; robert.adams@flinders.edu.au; 3 Central Adelaide Local Health Network, Adelaide, SA 5000, Australia; 4 CQUniversity Australia, The Appleton Institute, Wayville, SA 5034, Australia; a.reynolds@cqu.edu.au; 5 Adelaide Institute for Sleep Health: A Flinders Centre of Research Excellence, SA 5042, Australia

**Keywords:** actigraphy, cardiology, inpatients, readmissions

## Abstract

**Background:** Readmissions within 30 days of discharge are prominent among patients with cardiovascular disease. Post hospital syndrome hypothesizes that sleep disturbance during the index admission contributes to an acquired transient vulnerability, leading to increased risk of readmission. This study evaluated the association of in-hospital sleep (a) duration and (b) quality with 30-day all-cause unplanned readmission. **Methods:** This prospective observational cohort study included patients admitted to the coronary care unit of a South Australian hospital between 2016–2018. Study participants were invited to wear an ActiGraph GT3X+ for the duration of their admission and for two weeks post-discharge. Validated sleep and quality of life questionnaires, including the Pittsburgh Sleep Quality Index (PSQI), were administered. Readmission status and questionnaires were assessed at 30 days post-discharge via patient telephone interview and a review of hospital records. **Results:** The final cohort consisted of 75 patients (readmitted: *n* = 15, non-readmitted: *n* = 60), of which 72% were male with a mean age 66.9 ± 13.1 years. Total sleep time (TST), both in hospital (6.9 ± 1.3 vs. 6.8 ± 2.9 h, *p* = 0.96) and post-discharge (7.4 ± 1.3 h vs. 8.9 ± 12.6 h, *p* = 0.76), was similar in all patients. Patient’s perception of sleep, reflected by PSQI scores, was poorer in readmitted patients (9.13 ± 3.6 vs. 6.4 ± 4.1, *p* = 0.02). **Conclusions:** Although an association between total sleep time and 30-day readmission was not found, patients who reported poorer sleep quality were more likely to be readmitted within 30 days. This study also highlighted the importance of improving sleep, both in and out of the hospital, to improve the outcomes of cardiology inpatients.

## 1. Introduction

Hospital readmissions are an important indicator of healthcare safety and quality. Readmission within 30 days of discharge is a key quality metric reported to government organizations [1,2,3]. Up to 30% of readmissions occur within 30 days of discharge in patients diagnosed with a cardiovascular condition, predominantly in patients presenting with heart failure [4] and acute myocardial infarction [5]. Defining the risk factors of readmissions is important in determining optimal interventions to reduce readmissions [6,7,8] and improve patient outcomes.

Readmission risk prediction remains a complex and poorly understood endeavor which is currently dominated by patient-level models. These models include factors such as comorbidities, basic demographic data and clinical variables [9]. Moreover, no clearly defined predictors of readmissions have been noted in previous studies of cardiac cohorts [10,11,12]. Broader social, environmental, medical and functional factors are likely to contribute to readmission risk but have not been widely studied. Sleep disturbance is known to have negative physiological and psychological effects including altered emotions, poor memory, impaired cognitive function and reduced immunity [13]. The hospital environment is notorious for disrupting sleep [14], and sleep disturbance has been shown to interfere with healing [15]. A U-shaped relationship has been reported indicating that both short and long periods of sleep result in adverse cardiovascular outcomes [16,17]. Thus, poor sleep duration and quality in the hospital environment may be a factor contributing to readmissions.

Studies of sleep in hospitalized patients have been limited by the cohort sizes. This study uniquely focuses on a homogenous cohort of patients, unlike previously published studies [18,19], which have been based in general on medical cohorts with more heterogenous health complaints included for analysis. The association between sleep characteristics and readmission in cardiovascular inpatients has not been previously assessed. This study aims to determine if an association exists between sleep duration and sleep quality with 30-day all-cause unplanned readmission among cardiology inpatients, using both objective and self-reported measures of sleep. Given that poor sleep quality and duration is consistently associated with adverse metabolic outcomes, it is hypothesized that patients with shorter sleep duration and poorer sleep quality will be readmitted within 30 days.

## 2. Results

### 2.1. Patient Demographics

**Figure 1:**
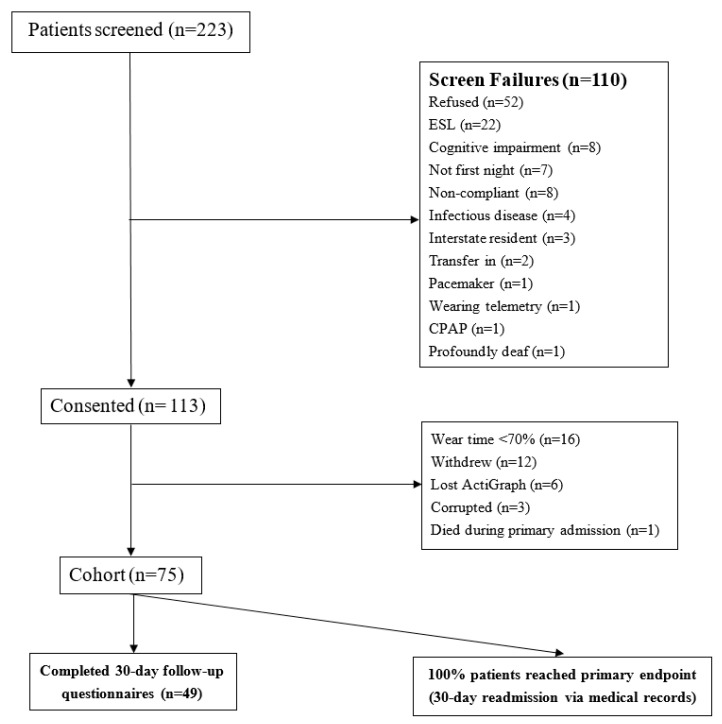
Consort diagram of study cohort. Abbreviations: ESL = English as a second language, CPAP = continuous positive airway pressure.

Of the 222 patients screened, 112 patients consented to participate, 12 patients withdrew and the remaining 26 did not participate, as reported in Figure 1. 

The remaining 75 patients formed the study cohort. The overall cohort were older adults (mean age of 66.9 ± 13.1 years) and the group was male-dominated (72%), with an average length of stay of 3.0 ± 1.5 days. Baseline characteristics of readmitted and non-readmitted patients were similar (Table 1). 

Comorbidities among all patients were similar, apart from the significantly greater number of readmitted patients presenting with a history of prior angina (73% vs. 25%, *p* = 0.03). Patient medications at admission were similar in all patients, although readmitted patients were more likely to be prescribed glyceryl trinitrate (GTN), consistent with their significant history of angina (33% vs. 13%, *p* = 0.07). The most common reason for the index admission was an elective procedure (33%) followed by acute coronary syndrome (27%) (Appendix B).

### 2.2. Readmission Timing and Causes

The 30-day follow-up rate for telephone calls was 65% (49 patients); however, readmission status was available via medical records for all 75 patients. The 30-day all-cause unplanned readmission rate was 20%. Time to readmission varied from one to 27 days post-discharge, with an average time of 11.9 ± 7.6 days. Patients wore an ActiGraph for an average of 2.8 ± 1.8 (range: 0 to 8) nights in hospital. Of the 37 patients who continued to wear the ActiGraph post-discharge, the average wear time was 10.0 ± 5.1 (range: 1 to 20) days. There were no clinical differences between patients who continued to wear the ActiGraph and those who did not (Appendix C). The most common cause of 30-day readmission was a cardiac diagnosis (nine of the 15 patients). A complete list of readmission diagnoses is available in Appendix D.

### 2.3. Objective Sleep Measures

In-hospital TST recordings showed no difference between readmitted and non-readmitted patients (6.9 ± 1.3 h vs. 6.8 ± 2.9 h, *p* = 0.96). A higher proportion of readmitted patients had longer TST (6–9 h vs. <6 h, *p* = 0.07) (Table 2). The post-discharge TST of non-readmitted patients was longer on average than readmitted patients; however, this was not statistically significant (7.4 ± 1.3 h vs. 8.9 ± 12.6 h, *p* = 0.76) (Table 2).

Wake After Sleep Onset (WASO) did not differ significantly between readmitted and non-readmitted patients, both in-hospital and post-discharge. However, in-hospital WASO recordings indicated that readmitted patients had longer WASOs compared to non-readmitted patients (84.5 ± 85.3 min vs. 61.9 ± 51.3 min, *p* = 0.14) with a medium effect size (Cohen’s d = 0.43). Further, when categorized, 67% of readmitted patients had a mean WASO of ≥60 min compared to 42% of non-readmitted patients (*p* = 0.16, Table 2). Although not statistically significant, in-hospital awakenings were increased in readmitted patients (13.6 ± 4.2 vs.11.9 ± 5.2, *p* = 0.25), with a small to medium effect size (Cohen’s d = 0.33) for the number of awakenings in hospital. There were no differences in the awake times between readmitted and non-readmitted patients for both the in-hospital and post-discharge data (Table 2).

### 2.4. Self-Reported Measures

No differences were observed between the readmitted and non-readmitted patients in relation to daytime sleepiness as measured by the Epworth Sleepiness Scale (ESS) (Table 3). The STOP BANG questionnaire scores were also similar between groups. However, 40% of readmitted patients (compared to 13% of non-readmitted patients, *p* = 0.02) reported that someone had observed them to stop breathing whilst sleeping.

The EQ-5D-3L was answered by all 75 patients at baseline, with readmitted patients reporting a lower VAS (Visual Analogue Scale) score (48.7 ± 21.9 vs. 63.3 ± 28.7, *p* = 0.07), indicative of poorer self-rated health, with a small effect size (Cohen’s d = 0.10). At 30 days, the mean VAS scores were similar between readmitted and non-readmitted patients (74.3 ± 15.1 vs. 76.5 ± 22.3, *p* = 0.80, respectively). The mobility domain of the EQ-5D-3L was significantly lower in readmitted patients at both baseline (*p* = 0.003) and 30 days (*p* = 0.004).

At baseline, readmitted patients had a lower PSQI global score, indicating worse sleep perception (9.13 ± 3.6 vs. 6.4 ± 4.1, *p* = 0.02). The average 30-day PSQI global scores were similar among readmitted patients and non-readmitted patients (6.3 ± 4.2 vs. 6.0 ± 3.9, *p* = 0.84).

## 3. Discussion

This is the first study to explore the association between sleep duration and quality with readmissions in cardiovascular inpatients. In line with previously published data [5], this study supports a significant rate of readmission in the first 30 days, with one in five patients returning to hospital. In relation to sleep, all patients slept for almost seven hours on average whilst in hospital, with a trend indicating that the majority of readmitted patients slept within the healthy range (6 to <9 h). On average, readmitted patients had a 20 min longer WASO in hospital, implying greater disruption during sleep. While a moderate effect size was observed, statistical significance was not reached, suggesting a need to further examine periods of wakefulness during hospital stays in a larger sample size. Similarly, readmitted patients had two more awakenings on average but spent less time awake during those awakenings. Evaluation of the ESS found higher than normal daytime sleepiness among all patients. The total scores for the STOP BANG found that all patients had an intermediate to high risk of obstructive sleep apnea (OSA). PSQI scores in all patients were greater than five at baseline and 30 days; thus, patients’ perception of their sleep quality was poor regardless of whether they were in or out of hospital. Importantly, the readmitted patients had a higher PSQI score at baseline, indicating that their recent sleep quality was noticeably poorer before their initial presentation compared to non-readmitted patients. Furthermore, readmitted patients had significantly more issues with mobility at baseline and at 30 days. This suggests that routinely unhealthy sleep may be associated with poorer quality of life and may be a contributor to a higher risk of readmission in patients with cardiovascular disease.

### 3.1. Sleep in the Inpatient Environment 

Sleep disruption during hospitalization is a significant concern for hospitalized patients. The negative consequences on patient health have been well described and result from a number of factors intrinsic to the patient, the external environment and the care process [20]. A systematic review of multiple interventions to limit the impact of sleep on hospitalized patients identified the potential to make improvements in the quality of patient sleep [21]. An example of a modifiable factor is noise levels in hospital, which can be improved through the provision of earplugs and eye masks. Changing the sound and light and even aroma in the environment has been shown to positively improve sleep in hospital [22]. Nursing care activities have also been implicated as the cause of sleep disruption, but efforts at limiting interventions have not been demonstrated to improve sleep conditions [23].

### 3.2. Sleep and Readmissions

The management of hospitalized patients is focused on the acute illness that led to the admission. There is often little focus on managing stressors during hospitalization that may contribute to the vulnerabilities and possible triggers that may lead to readmission. Changes to sleep, physical inactivity, social isolation, dietary changes, and modifications in ambient brightness and temperature during hospitalization produce responses observed as delirium, immunosuppression and depression.

**Figure 2:**
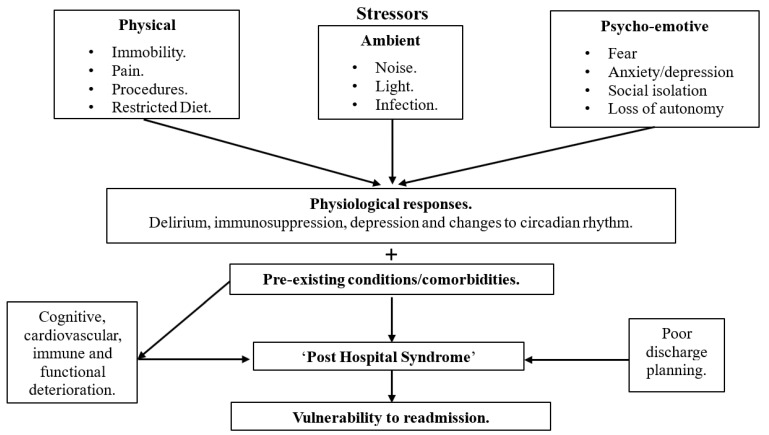
Possible mechanisms behind post-hospital syndrome that result in patient readmission. Adapted from Figure 1 (Mesquita et al. 2015) [26]. Sleep is a stressor that is partly physical, partly ambient and partly psycho-emotive. These in-hospital stressors result in physiological responses. In addition to pre-existing comorbidities, the cognitive, cardiovascular, immune and functional deterioration and poor discharge planning lead to an increased risk of post hospital syndrome. This then leads to patients having increased vulnerability to readmission.

The consequence of the myriad of potential contributors have been expressed in the literature as post hospital syndrome [24], which hypothesizes that the index admission increases a patient’s generalized risk and vulnerability for readmission [24]. As the index illness begins to heal and the patient is discharged, post hospital syndrome manifests and leads to readmission. The exact etiology of post hospital syndrome is not well understood; however, allostasis overload during the index hospitalization has been postulated [25], whereby the increased level of chronic stress can lead to cognitive, cardiovascular, immune and functional deterioration. These mechanisms are depicted in Figure 2 [26]. Sleep disturbance during the index admission may be one of several factors contributing to a patient’s vulnerability to readmission [24], and it is well established that acute sleep deprivation causes disruptions to the circadian rhythm [27]. The long-term impacts of disturbed sleep during hospitalization include lower physical functioning, increased mortality and delirium [21]; however, there are little to no data specifically assessing sleep in cardiovascular inpatients and the impact on readmissions.

Our current understanding of sleep in cardiovascular patients is focused on OSA, with observational evidence showing that OSA is associated with both coronary and cerebrovascular morbidity and mortality [28,29]. This study showed eight (11%) patients from the overall cohort had been previously diagnosed with OSA (Table 1). However, 14 patients (19%) of the overall cohort indicated that someone had observed them to stop breathing while sleeping, suggesting a potential under-diagnosis of OSA among cardiology patients. Additionally, a recent study in a population of patients with mild OSA symptoms using coronary computed tomography angiography found no significant correlation between calcification measured by the calcium artery score and OSA [30]. The study did, however, find an association between calcification and OSA, when calcification was measured by segment involvement score and segment stenosis score. A US study of general medicine patients reported OSA as a risk factor for readmission (11.4% vs. 7.6%, *p* < 0.01) [31]. Thus, OSA may be an important novel modifiable risk factor for cardiovascular readmissions. A previously published study [32] found that patients who were compliant with CPAP had a reduced 30-day cardiac readmission rate. This was unable to be assessed, or controlled for, in the present study, as no patients reported actively using CPAP.

A meta-analysis of sleep duration (assessed by various methods) in cardiovascular cohorts demonstrated that less than five hours and over nine hours of sleep was associated with poorer outcomes; specifically, mortality and morbidity. However, none of the studies in the meta-analysis assessed readmission [33]. Few studies have compared sleep in-hospital to at home; moreover, no data have been reported in a cardiovascular cohort. A large Dutch cross-sectional observational study [34] of 2000 general medicine and surgical inpatients assessed duration and quality of sleep and found that, on average, patients slept for 83 min less in hospital. This study also found more nocturnal awakenings in hospital than at home, similar to the findings of the current study. An Australian study assessing the perceived duration of sleep in generally hospitalized patients also found that patients slept less in hospital than at home [35]. Both studies assessed sleep using self-reported measures, and thus this study presents the first objective measurement of sleep using actigraphy for hospitalized patients.

### 3.3. Limitations

The major limitation of this study was the small sample size, illustrated well by the reasonable effect sizes between readmitted and non-readmitted patients; however, these results did not achieve statistical significance. Importantly, this study demonstrates the feasibility of using wearable devices for hospitalized patients, although post-discharge use may require more monitoring for adherence. It should be noted that the ActiGraph was not consistently worn on either the dominant or non-dominant wrist due to the clinical setting, and this may have confounded the results. The small sample size also limited our ability to adjust for potential confounders, such as comorbidities which may be related to readmissions. This study was limited due to the lack of a sleep diary, which has been previously shown to corroborate the findings of actigraphy, particularly for non-compliant wear and when a patient is motionless but still awake [36]. Hospital-based actigraphy has been shown to over-estimate sleep duration and efficacy, whilst underestimating the amount of wake time [37,38]. These limitations have been highlighted in intensive care unit patients [39,40]; for example, the lack of movement in ventilated or sedated patients leads to an over-estimation of sleep. While these limitations should be acknowledged, the inclusion of actigraphy meant that reliance on self-reporting was avoided, and the study could be further strengthened in future by considering ambulatory polysomnography. The duration and number of daytime naps were not independently analyzed in this study, and future studies should consider the role of napping behavior. This study enrolled patients with any cardiovascular diagnosis, and to improve generalizability, future studies may be limited to a specific cardiovascular condition such as acute myocardial infarction or heart failure. Although there is limited literature available to date on sleep disorders and readmissions, future studies may investigate whether patients—either with treated or untreated sleep disorders—have a higher risk of readmissions. The inclusion criteria of one night of admission may have been too short to adversely impact sleep compared to the home environment. Future studies with larger sample sizes should account for duration of stay as a covariate in order to establish any potential impact. Additionally, future studies may explore the environmental stressors which may be a contributing factor to the findings of this study.

## 4. Methods and Materials

This prospective observational cohort study recruited patients spending a minimum of one night in the coronary care unit of The Queen Elizabeth Hospital, South Australia between June 2016 and March 2018. This study was approved by Central Adelaide Local Health Network (CALHN) Human Research Ethics Committee.

### 4.1. Study Patients

The inclusion criteria for this study were (i) hospital admission with a primary cardiovascular diagnosis or procedure and (ii) the ability to wear an ActiGraph device on the wrist. Patients were excluded if they (i) were not residing permanently within South Australia, (ii) had a movement disorder, (iii) were highly dependent on medical care based on discussion with nursing staff for example patients who were in a coma or intubated, (iv) were assessed by ward staff as being cognitively or intellectually unable to participate or (v) were unable to provide informed consent or communicate sufficiently in English.

### 4.2. Study Protocol

Following informed consent and prior to the patient’s first night in hospital, questionnaires assessing sleep and health status were administered, including the Pittsburgh Sleep Quality Index (PSQI) [41], EuroQoL-5 Dimensions-3 Levels (EQ-5D-3L) [42], Epworth Sleepiness Scale (ESS) [43] and the STOP BANG [44] questionnaire (a score based on patient snoring, tiredness, observed apnea, blood pressure, body mass index, age, neck circumference and gender). Medical history was attained via patient interview and medical record review. The ActiGraph GT3X+ (ActiGraph, LLC, Pensacola, FL) was placed on the patient’s preferred wrist, taking into consideration the placement of any medical devices (such as cannulas and tubing), and the patient was instructed to wear it continuously. Patients were invited to continue wearing the ActiGraph for two weeks following discharge and provided with a post-paid reply envelope to return the device. If the patient preferred to return the ActiGraph at discharge, the ActiGraph was collected by the researcher following discharge.

### 4.3. 30-Day All-Cause Unplanned Readmission

A readmission was defined as a return to the emergency department or an unplanned admission to hospital for any reason within 30 days of the patient’s discharge. Readmissions were determined via hospital administrative databases and medical records or from patient self-report at the 30-day follow-up.

### 4.4. Follow-Up

At 30 days post-discharge, patients were contacted via telephone or mail following two failed telephone attempts. Patients were asked whether they had returned to hospital since discharge, and both the PSQI [41] and EQ-5D-3L [42] questionnaires were reassessed.

### 4.5. Sleep Parameters

#### 4.5.1. Actigraphy

Polysomnography (PSG) is the gold standard measure of sleep duration; however, due to the intrusive nature of the device [45], it was not feasible for use in this cohort of patients. Actigraphy uses a piezoelectric transducer to measure wake/sleep state based on movement [46]. The ActiGraph GT3X+ has been validated in various cohorts against laboratory-based PSG [47,48,49,50] with 90% sensitivity, 46% specificity and 84% accuracy [51]. All ActiGraph data was extrapolated using ActiLife version 6.0 software (ActiGraph LLC).

The Troiano algorithm [52] was used to interpret sleep measures. A wear time threshold has not been previously defined in the literature, and various thresholds were therefore assessed for the total sleep time (TST) of all patients who wore an ActiGraph. An average 70% threshold was determined to be sufficient for the data analysis of the cohort (refer to Appendix A for a full description). The Cole–Kripe sleep algorithm [53] was used to interpret the ActiGraph data into the following endpoints:

Total sleep time (TST): The average time (minutes) a patient was asleep during a 24-h period.

Number of awakenings: The average number of times the patient awoke during a period characterized as sleep.

Wake After Sleep Onset (WASO): The average time (minutes) between sleep and wake. In healthy sleep, WASO should be <5% of TST [54].

Average time awake: The average amount of time (minutes) the patient was awake during a period of sleep.

#### 4.5.2. Pittsburgh Sleep Quality Index (PSQI) 

The PSQI is a validated measure of a patient’s perception of their sleep and is comprised of 19 questions and seven domains [41,55]. The global score is ranked from 0 to 21, where scores ≥5 are defined as poor sleep with 90% sensitivity and 87% specificity compared to PSG [41].

#### 4.5.3. Epworth Sleepiness Scale (ESS) 

The ESS assesses the respondent’s propensity to fall asleep during the day across a range of daytime activities and scenarios [43]. The ESS is comprised of eight questions, with higher scores indicating increased daytime sleepiness [43,55,56]. The ESS has shown to have high internal consistency (Cronbach alpha = 0.88) among healthy medical students [56].

#### 4.5.4. STOP BANG

The STOP BANG questionnaire [44] is comprised of eight items and has been validated against PSG. It detects obstructive sleep apnea (OSA) for scores ≥5, with 72% (CI 54.4–89.6) sensitivity and 33.3% (CI 2.5–64.1) specificity [57].

#### 4.5.5. EuroQoL-5 Dimensions- 3 Levels (EQ-5D-3L) 

The EQ-5D-3L is a well-established quality of life questionnaire [42] with acceptable construct validity [58] and excellent reproducibility [59].

### 4.6. Statistical Analyses

Descriptive data for the readmitted and non-readmitted patients were analyzed using t-tests (for continuous variables) and chi^2^ tests (for categorical variables). Mean TST was displayed as both a continuous variable and categorized variable, classified into <6 h [60,61,62,63], 6 to 9 h and >9 h, as has been reported previously [62,63,64,65,66,67,68,69,70]. Effect size was measured using Cohen’s d test to determine whether a magnitude or size difference exists between the mean pre and post discharge values. All statistical analyses were performed using STATA 14 (StataCorp., College Station, TX, USA).

## 5. Conclusions

This study did not find a statistically significant relationship between sleep duration (as measured by actigraphy) and 30-day all-cause unplanned readmissions among cardiology inpatients. However, poorly perceived sleep quality (measured by the PSQI [41]) was associated with increased 30-day all-cause unplanned readmissions. Whilst the actigraphy data was not associated with readmissions, some aspects of sleep in the readmitted patients, including the WASO and in-hospital awakenings, suggest that further exploration is warranted in larger studies. This may have implications for inpatient management if disturbance of sleep is linked to readmissions. If so, future research involving risk prediction models for hospital readmissions may be improved by wearable device technology to monitor and collect sleep data. Finally, it is important to characterize how changes in sleep pattern during hospitalization correlate with physiological abnormalities that may increase the risk of adverse outcomes after hospitalization.

## Figures and Tables

**Table 1:** Baseline characteristics for unplanned readmitted vs. non-readmitted patients.

	Readmitted		Not Readmitted		*p*
	Mean	SD	Mean	SD	
***Demographics and Comorbidities***					
*n*	15	-	60	-	-
Age (years)	64.5	18.7	67.5	11.5	0.43
Sex (% Females)	40	-	25	-	0.25
Length of Stay (days)	3.5	1.5	2.8	1.5	0.14
Private insurance (%)	47	-	38	-	0.57
Single room (%)	33	-	30	-	0.80
Live alone (%)	47	-	28	-	0.17
***Cardiovascular risk factors and comorbidities***					
AF (%)	33	-	32	-	0.90
HF (%)	33	-	15	-	0.10
Dyslipidemia (%)	67	-	68	-	0.90
Hypertension (%)	60	-	75	-	0.25
Prior Stroke (%)	20	-	12	-	0.40
Prior Diabetes (%)	20	-	32	-	0.38
Prior Angina (%)	73	-	25	-	0.03
Prior MI (%)	33	-	18	-	0.21
Prior PCI (%)	13	-	22	-	0.47
Prior CABG (%)	20	-	8	-	0.19
PAD (%)	13	-	3	-	0.13
Current smoker (%)	20	-	23	-	0.95
***Non-Cardiovascular risk factors or comorbidities***					
COPD (%)	20	-	13	-	0.51
Arthritis (%)	13	-	22	-	0.47
Depression (%)	20	-	10	-	0.29
Anxiety (%)	7	-	5	-	0.81
No OSA (%)	87	-	90	-	0.84
OSA with CPAP (%)	7	-	7	-	
OSA w/o CPAP (%)	7	-	3	-	
Asthma (%)	20	-	12	-	0.40
GORD (%)	13	-	30	-	0.19
***Procedures and medications***					
Angiography (%)	27	-	47	-	0.15
Aspirin (%)	33	-	48	-	0.30
Statin (%)	40	-	50	-	0.49
ARB / ACE inhibitor (%)	40	-	42	-	0.91
GTN (%)	33	-	13	-	0.07

**Abbreviations:** AF = atrial fibrillation; HF = heart failure; PCI= percutaneous coronary intervention; CABG = coronary artery bypass graft; AAA = abdominal aortic aneurysm; MI = myocardial infarction; OSA = obstructive sleep apnea; CPAP = continuous positive air pressure; GORD = gastro-esophageal reflux disease; PAD = peripheral artery disease; COPD = chronic obstructive pulmonary disease; ARB = angiotensin II receptor blocker; ACE = angiotensin converting enzyme; GTN = glyceryl trinitrate. *p* values were taken from *t*-test or chi^2^ test, as appropriate.

**Table 2:** In-hospital and post-discharge ActiGraph data.

	Readmitted		Not Readmitted		*p*	Cohen’s d (95%CI)
	Mean	SD	Mean	SD		
**In hospital**						
*Total sleep time* (n)	14	-	59	-	-	-
In hours	6.9	1.3	6.8	2.9	0.96	0.02 (−0.57–0.59)
0 to 6 h (%)	21	-	44	-	0.07	-
6 to <9 h (%)	71	-	37	-	-	-
≥ 9 h (%)	7	-	19	-	-	-
*Wake AfterSleep Onset* (n)	15	-	59	-	-	-
*In minutes*	84.5	85.3	61.9	51.3	0.14	0.43 (−0.14–1.00)
<30 min (%)	0	-	12	-	0.16	-
30 to 60 min (%)	33	-	46	-	-	-
≥ 60 min (%)	67	-	42	-	-	-
*Number of awakenings* (n)	15	-	59	-	-	*-*
	13.6	4.2	11.9	5.2	0.25	0.33 (−0.23–0.91)
*Average time awake* (n)	15	-	59	-	-	-
In minutes	4.7	1.6	5.4	2.4	0.29	0.30 (−0.26–0.87)
**Post-discharge**						
*Total sleep time* (n)	7	-	30	-	-	-
Hours	7.4	1.3	8.9	12.6	0.76	0.13 (−0.69–0.94)
0 to 6 h (%)	14.3	-	33	-	0.54	-
6 to <9 h (%)	71.4	-	60	-	-	-
≥ 9 h (%)	14.3	-	7	-	-	-
*Wake AfterSleep Onset* (n)	7	-	31	-	-	-
In minutes	43.4	15.6	46.2	14.6	0.65	0.19 (−0.63–1.01)
<30 min (%)	14	-	16	-	0.78	-
30 to 60 min (%)	72	-	58	-	-	-
≥ 60 min (%)	14	-	26	-	-	-
*Number of awakenings* (n)	7	-	31	-	-	-
	11.1	5.5	11.2	4.3	0.80	0.10 (−0.71–0.92)
*Average time awake* (n)	7	-	31	-	-	-
In minutes	5.6	2.8	4.9	1.7	0.47	0.31 (−0.51–1.13)

*p* values were taken from *t*-test or chi^2^ test, as appropriate. 95%CI: 95% confidence interval.

**Table 3:** Sleep and Quality of Life Questionnaires data (in-hospital and post-discharge).

	Readmitted		Not Readmitted		*p*	Cohen’s d (95%CI)
	Mean	SD	Mean	SD		
***In-hospital***						
n	15	-	60	-	-	-
ESS Mean Score	5.9	5.3	6.3	4.6	0.73	0.09 (−0.47–0.66)
STOP BANG	4.3	1.5	4.1	1.5	0.70	0.11 (−0.45–0.67)
PSQI	9.13	3.6	6.4	4.1	0.02	0.70 (0.12–1.27)
n	15	-	58	-	-	-
EQ-5D VAS	48.7	21.9	63.3	28.7	0.07	0.53 (−0.04–1.11)
***Post-discharge***						
n	7	-	40	-	-	-
EQ-5D VAS	74.3	15.1	76.5	22.3	0.80	0.10 (−0.70–0.91)
n	8	-	41	-	-	-
PSQI	6.3	4.2	6.0	3.9	0.84	0.08 (−0.68–0.83)

*p* values were taken from t-test or chi^2^ test, as appropriate. ESS: Epworth Sleepiness Scale; PSQI: Pittsburgh Sleep Quality Index; VAS: Visual Analogue Scale.

## Appendix A. Validity of ActiGraph Wear Time

**Table A1:** For all patients who wore an ActiGraph (*n* = 92), regardless of the wear time.

Average Time Worn (%)	Number of Patients	% of Patients
0	1	1.09
4.4	1	1.09
41.1	1	1.09
42.8	1	1.09
44	1	1.09
44.7	1	1.09
44.9	1	1.09
46.3	1	1.09
51.8	1	1.09
59.5	1	1.09
67	3	3.26
67.8	1	1.09
70.3	1	1.09
74	2	2.17
74.3	1	1.09
74.4	1	1.09
75	1	1.09
75.5	1	1.09
77	1	1.09
77.4	1	1.09
78.4	1	1.09
78.8	1	1.09
79.3	1	1.09
80	2	2.17
80.4	1	1.09
81.6	1	1.09
82	2	2.17
83.5	1	1.09
84.3	1	1.09
84.3	1	1.09
84.5	1	1.09
84.7	1	1.09
85.4	1	1.09
85.5	1	1.09
86	1	1.09
86.5	1	1.09
86.6	1	1.09
87	1	1.09
87.4	1	1.09
88.3	1	1.09
88.7	1	1.09
88.8	1	1.09
89	1	1.09
89.5	1	1.09
90	1	1.09
91.3	1	1.09
91.4	1	1.09
91.6	1	1.09
91.9	1	1.09
92	4	4.35
93.2	1	1.09
94.6	1	1.09
94.8	1	1.09
96.3	1	1.09
96.5	1	1.09
96.8	1	1.09
97.4	1	1.09
97.7	1	1.09
97.9	1	1.09
98	1	1.09
98.6	1	1.09
98.8	1	1.09
98.95	1	1.09
NA	3	3.26
100	18	19.6
**Total**	**92**	

Three patients had a value of NA as a wear time—this was because the data did not have useable or interpretable data. Thus, there were 89 patients with ActiGraph data that were recorded with wear times wearing from 0% to 100% wear time.

From these data, we lowered the average wear time to ≥80% (there were 63 patients), as shown in Table 2.

**Table A2:** (**a**) Patients who had a wear time ≥80% (*n* = 63). (**b**) Differences between patients with ≥80% wear time and <80% wear time. Simple t-test and chi^2^ tests were performed to determine a *p* value.

**(a)**			
**Average Time Worn (%)**	**Number of Patients**		**% of Patients**
80	2		2.2
80.4	1		1.1
81.6	1		1.1
82	2		2.2
83.5	1		1.1
84.3	1		1.1
84.3	1		1.1
84.5	1		1.1
84.7	1		1.1
85.4	1		1.1
85.5	1		1.1
86	1		1.1
86.5	1		1.1
86.6	1		1.1
87	1		1.1
87.4	1		1.1
88.3	1		1.1
88.7	1		1.1
88.8	1		1.1
89	1		1.1
89.5	1		1.1
90	1		1.1
91.3	1		1.1
91.4	1		1.1
91.6	1		1.1
91.9	1		1.1
92	4		4.4
93.2	1		1.1
94.6	1		1.09
94.8	1		1.09
96.3	1		1.09
96.5	1		1.09
96.8	1		1.09
97.4	1		1.09
97.7	1		1.09
97.9	1		1.09
98	1		1.09
98.6	1		1.09
98.8	1		1.09
98.95	1		1.09
100	18		19.6
**Total**	63		
**(b)**			
	**Wear Time (%)**		
	**<80% (*n* = 29)**	**≥80% (*n* = 63)**	***p***
Age	71±12.1	66.5±13.3	0.1246
Female	11/29 =	15/63 =	0.162
ESS baseline	6.7±4.2	6.1±4.7	0.5964
EQ5D baseline	58.8±26.5	58.9±28.4	0.9789
PSQI baseline	5.2±3.1	7.1±4.2	0.0371
STOP BANG baseline	4.1±1.4	4.1±1.5	0.9415
Body Mass Index	28.1±5.6	30.1±5.4	0.1138
Neck circumference	39.4±2.5	39.2±4.1	0.8496
Length of stay	3.2±2.7	3.0±1.5	0.6345

**Table A3:** (**a**) Patients who had a wear time ≥75% (*n* = 70). (**b**) Differences between patients with ≥75% wear time and <75% wear time. Simple t-test and chi^2^ tests were performed to determine a *p* value.

**(a)**			
**Average Time Worn (%)**	**Number of Patients**		**% of Patients**
75	1		1.09
75.5	1		1.09
77	1		1.09
77.4	1		1.09
78.4	1		1.09
78.8	1		1.09
79.3	1		1.09
80	2		2.17
80.4	1		1.09
81.6	1		1.09
82	2		2.17
83.5	1		1.09
84.3	1		1.09
84.3	1		1.09
84.5	1		1.09
84.7	1		1.09
85.4	1		1.09
85.5	1		1.09
86	1		1.09
86.5	1		1.09
86.6	1		1.09
87	1		1.09
87.4	1		1.09
88.3	1		1.09
88.7	1		1.09
88.8	1		1.09
89	1		1.09
89.5	1		1.09
90	1		1.09
91.3	1		1.09
91.4	1		1.09
91.6	1		1.09
91.9	1		1.09
92	4		4.35
93.2	1		1.09
94.6	1		1.09
94.8	1		1.09
96.3	1		1.09
96.5	1		1.09
96.8	1		1.09
97.4	1		1.09
97.7	1		1.09
97.9	1		1.09
98	1		1.09
98.6	1		1.09
98.8	1		1.09
98.95	1		1.09
100	18		19.6
**Total**	70		
**(b)**			
	**Wear Time (%)**		
	**<75% (*n* = 22)**	**≥75% (*n* = 70)**	***p***
Age	71.3 ± 13.3	66.8 ± 12.9	0.1620
Female	10	16	0.040
ESS baseline	6.5 ± 4.2	6.2 ± 4.7	0.8401
EQ5D baseline	55.2 ± 29.1	60.0 ± 27.3	0.4955
PSQI baseline	4.6 ± 2.7	7.1 ± 4.2	0.0101
STOP BANG baseline	3.9 ± 1.4	4.1 ± 1.5	0.5125
Body Mass Index	28.8 ± 6.1	29.7 ± 5.3	0.4895
Neck circumference	39.5 ± 2.8	39.2 ± 3.9	0.7898
Length of stay	3.5 ± 3.1	3.0 ± 1.4	0.3281

**Table A4:** (**a**) Patients who had a wear time ≥70% (*n* = 75). (**b**) Differences between patients with ≥70% wear time and <70% wear time. Simple t-test and chi^2^ tests were performed to determine a *p* value.

**(a)**			
**Average Time Worn (%)**	**Number of Patients**		**% of Patients**
70.3	1		1.09
74	2		2.17
74.3	1		1.09
74.4	1		1.09
75	1		1.09
75.5	1		1.09
77	1		1.09
77.4	1		1.09
78.4	1		1.09
78.8	1		1.09
79.3	1		1.09
80	2		2.17
80.4	1		1.09
81.6	1		1.09
82	2		2.17
83.5	1		1.09
84.3	1		1.09
84.3	1		1.09
84.5	1		1.09
84.7	1		1.09
85.4	1		1.09
85.5	1		1.09
86	1		1.09
86.5	1		1.09
86.6	1		1.09
87	1		1.09
87.4	1		1.09
88.3	1		1.09
88.7	1		1.09
88.8	1		1.09
89	1		1.09
89.5	1		1.09
90	1		1.09
91.3	1		1.09
91.4	1		1.09
91.6	1		1.09
91.9	1		1.09
92	4		4.35
93.2	1		1.09
94.6	1		1.09
94.8	1		1.09
96.3	1		1.09
96.5	1		1.09
96.8	1		1.09
97.4	1		1.09
97.7	1		1.09
97.9	1		1.09
98	1		1.09
98.6	1		1.09
98.8	1		1.09
98.95	1		1.09
100	18		19.6
**Total**	75		
**(b)**			
	**Wear Time (%)**		
	**<70% (*n* = 17)**	**≥70% (*n* = 75)**	***p***
Age	71.8 ± 13.5	67.0 ± 12.9	0.1727
Female	8	18	0.057
ESS baseline	6.1 ± 4.2	6.3 ± 4.7	0.8694
EQ5D baseline	49.1 ± 28.6	61.1 ± 27.1	0.1069
PSQI baseline	3.9 ± 2.0	7.1 ± 4.0	0.0031
STOP BANG baseline	3.7 ± 1.4	4.2 ± 1.5	0.2321
Body Mass Index	28.3 ± 6.7	29.8 ± 5.2	0.3420
Neck circumference	38.9 ± 2.8	39.3 ± 3.8	0.6807
Length of stay	3.8 ± 3.5	2.9 ± 1.4	0.0894

## Appendix B

**Table A5:** Cohort Primary Diagnoses.

Diagnosis Category	*n*	%
Elective procedures	22	33
Acute coronary syndromes	18	27
Arrhythmias	8	12
Heart failure	7	11
Other	11	17

Note all patients who came in with chest pain were listed under ‘other’.

## Appendix C

**Table A6:** Patient characteristic comparison of those who continued and to wear the ActiGraph post-discharge and those who did not.

	Wore ActiGraph Post-Discharge		Did Not Wear ActiGraph Post-Discharge		*p*
	Mean	SD	Mean	SD	
***Demographics and Comorbidities***					
*n*	37	-	38	-	-
Age (years)	67.4	14.4	66.4	12.0	0.75
Sex (% Females)	37.8	-	18.4	-	0.06
Length of Stay (days)	2.9	1.4	3.0	1.6	0.93
Private insurance (%)	37.8	-	42.1	-	0.71
Atrial Fibrillation (%)	37.8	-	26.3	-	0.29
Heart Failure (%)	13.5	-	23.7	-	0.26
Dyslipidemia (%)	70.3	-	65.8	-	0.68
Hypertension (%)	70.3	-	73.7	-	0.74
Prior Stroke (%)	16.2	-	10.5	-	0.47
Prior Diabetes (%)	32.4	-	26.3	-	0.56
Prior Angina (%)	40.5	-	55.3	-	0.20
Prior Myocardial Infarction (%)	16.2	-	26.3	-	0.26
Prior Percutaneous Coronary Intervention (%)	24.3	-	15.8	-	0.36
Current Smoker (%)	21.6	-	23.7	-	0.28
Ischemic Heart Disease (%)	40.5	-	44.7	-	0.71
Chronic Obstructive Pulmonary Disease (%)	10.8	-	18.4	-	0.35

## Appendix D

Of the 15 patients who returned to hospital, the causes of readmission are listed below: nine of 15 were readmissions for something related to the cardiovascular system.

**Table A7:** Causes of Readmission.

Cardiovascular-Related Readmission	Non-Cardiovascular-Related Readmission
**ST-elevated myocardial infarction**	Return to Emergency Department for unknown reason (x2)
**Exacerbation of heart failure**	Gynaecological pain
**Coronary spasm**	Haematoma on the arm
**Chest pain (x3)**	Itchy skin rash
**Atrial fibrillation**	Accidental overdose
**Atypical angina**	
**Unstable angina**	
